# Does women’s age matter in the SDGs era: coverage of demand for family planning satisfied with modern methods and institutional delivery in 91 low- and middle-income countries

**DOI:** 10.1186/s12978-020-0903-6

**Published:** 2020-04-19

**Authors:** Inacio Crochemore M. da Silva, Fernanda Everling, Franciele Hellwig, Carine Ronsmans, Lenka Benova, Jennifer Requejo, Anita Raj, Aluisio J. D. Barros, Cesar G. Victora

**Affiliations:** 1 International Center for Equity in Health, Post-graduate Program in Epidemiology, Federal University of Pelotas, Marechal Deodoro, 1160, Pelotas, RS 96020-220 Brazil; 2 Faculty of Epidemiology and Population Health, London School of Hygiene and Tropical Medicine, London, UK; 3 Institute of Tropical Medicine, Antwerp, Belgium; 4 Health and HIV Division of Planning, Analysis and Monitoring, UNICEF, New York, NY USA; 5 Center on Gender Equity and Health, Department of Medicine, University of California San Diego, San Diego, USA; 6 Department of Education Studies, University of California San Diego, San Diego, USA

**Keywords:** Health inequalities, Age patterns, Reproductive health, Sustainable development goals

## Abstract

**Background:**

The Sustainable Development Goals (SDGs) include specific targets for family planning (SDG 3.7) and birth attendance (SDG 3.1.2), and require analyses disaggregated by age and other dimensions of inequality (SDG 17.18). We aimed to describe coverage with demand for family planning satisfied with modern methods (DFPSm) and institutional delivery in low- and middle-income countries across the reproductive age spectrum. We attempted to identify a typology of patterns of coverage by age and compare their distribution according to geographic regions, World Bank income groups and intervention coverage levels.

**Methods:**

We used Demographic and Health Survey and Multiple Indicator Cluster Surveys. For DFPSm, we considered the woman’s age at the time of the survey, whereas for institutional delivery we considered the woman’s age at birth of the child. Both age variables were categorized into seven groups of 5 year-intervals, 15–19 up to 45–49. Five distinct patterns were identified: (a) increasing coverage with age; (b) similar coverage in all age groups; (c) U-shaped; (d) inverse U-shaped; and (e) declining coverage with age. The frequency of the five patterns was examined according to UNICEF regions, World Bank income groups, and coverage at national level of the given indicator.

**Results:**

We analyzed 91 countries. For DFPSm, the most frequent age patterns were inverse U-shaped (53%, 47 countries) and increasing coverage with age (41%, 36 countries). Inverse-U shaped patterns for DFPSm was the commonest pattern among lower-middle income countries, while low- and upper middle-income countries showed a more balanced distribution between increasing with age and U-shaped patterns. In the first and second tertiles of national coverage of DFPSm, inverse U-shaped was observed in more than half of countries. For institutional delivery, declining coverage with age was the prevailing pattern (44%, 39 countries), followed by similar coverage across age groups (39%, 35 countries). Most (79%) upper-middle income countries showed no variation by age group while most low-income countries showed declining coverage with age (71%).

**Conclusion:**

Large inequalities in DFPSm and institutional delivery were identified by age, varying from one intervention to the other. Policy and programmatic approaches must be tailored to national patterns, and in most cases older women and adolescents will require special attention due to lower coverage and because they are at higher risk for maternal mortality and other poor obstetrical outcomes.

## Plain English summary

This paper examined inequalities in demand for family planning satisfied with modern methods (DFPSm) and institutional delivery in low- and middle-income countries across the reproductive age spectrum. Both indicators analyzed are closely linked to the Sustainable Development Goals (SDGs). Family planning is mentioned in the goal for universal access to sexual and reproductive health (SDG 3.7), while institutional delivery closely correlates with SDG 3.1.2. on coverage with skilled attendance at birth. In addition, emphasizing the need to leave no one behind, SDG 17.18 requires general analyses disaggregated by age and other types of inequality. Using 91 Demographic and Health Survey and Multiple Indicator Cluster Surveys five distinct patterns were identified: (a) increasing coverage with woman’s age; (b) similar coverage in all age group; (c) U-shaped; (d) inverse U-shaped; and (e) declining coverage with woman’s age group. Large inequalities in DFPSm and institutional delivery were identified according to the age of the women. For DFPSm, the most frequent age-related patterns were inverse U-shaped and increasing coverage with age. Inverse-U shaped was most frequent among lower-middle income countries, while low-income and upper middle-income countries showed similar frequencies of the increasing with age and U-shaped patterns. For institutional delivery, declining coverage with age was the prevailing pattern, followed by similar coverage across age groups. Most upper-middle income countries showed no variation by age, while most low-income countries showed declining coverage with age. Policy and programmatic approaches must be tailored to national patterns considering the differences between indicators. In most cases, older women and adolescents will require special attention due to lower coverage levels and because they are at higher risk for maternal mortality and other poor obstetrical outcomes.

## Introduction

The Sustainable Development Goals (SDG) include specific targets for maternal (SDG 3.1: reduce the global maternal mortality ratio to less than 70 per 100,000 live births) and neonatal mortality (SDG 3.2: reduce neonatal mortality to 12 per 1000 live births and under-5 mortality to 25 per 1000 live births). Reaching these targets requires a greater focus on family planning, antenatal and delivery care interventions.

Critics of the Millennium Development Goal (MDG) targets [1, 2] pointed out that, rather than focusing solely on national estimates, monitoring efforts should report on within-country inequalities, as adequate progress at national level is possible even when subgroups of the population fail to make progress. This omission was addressed by the (SDGs) with their emphasis on leaving no one behind, in particular with SDG 17.18, which requires disaggregation of indicators by income, gender and age, among dimensions of inequality. While equity indicators such as poverty, rural residence and low maternal education are well-documented risk factors for maternal and neonatal mortality as well as lower utilization of health care services [3], age as an equity variable has received less attention. Yet, previous studies have shown that women above age 30 are at greater risk for maternal mortality [4] and children of adolescent girls at greatest risk for neonatal mortality [5], relative to women in their 20s. More recently, disaggregation by age of the woman has focused on intervention coverage among adolescent girls compared to older women [6–8]. Less is known, however, on how intervention coverage is related to woman’s ages across the whole spectrum of age ranges, from 15 to 49 years.

We report on a multi-country analysis of coverage with interventions by women’s age, focusing on two key indicators of maternal health: demand for family planning satisfied with modern methods (DFPSm) and institutional delivery. Both indicators are closely related to the SDGs. Family planning is one aspect of the targets around universal access to sexual and reproductive health found in the SDGs 3.7 and 5.6. Institutional delivery closely correlates with SDG 3.1.2. on coverage with skilled attendance at birth; a recent analysis showed a correlation of 0.956 (*P* < 0.001) among the two indicators at national level [9]. In addition, institutional delivery is less affected by issues regarding definition at national level, which is the case for skilled birth attendance with the definition of skilled providers. There is strong evidence that use of effective family planning methods and institutional delivery contribute to reducing maternal and newborn deaths [10–13]. Further, engagement in these behaviors appear to vary generationally and by age, with adolescent and older women theoretically less likely to utilize both services in many national contexts [14, 15].

The present study aimed to describe coverage with DFPSm and institutional delivery in low- and middle-income countries across the reproductive age spectrum, using recent national surveys. We attempted to identify a typology of patterns of coverage by age and compare the distribution of such patterns according to the countries’ geographic regions, World Bank income groups and intervention coverage levels.

## Methods

We used Demographic and Health Survey (DHS) and Multiple Indicator Cluster Surveys (MICS) [16, 17] to identify and compare coverage levels of the two indicators in low- and middle-income countries (LMICs). Both types of surveys are performed with similar methods, using multi-stage cluster procedures to select representative samples of women of reproductive age (15–49 years) and children under 5 years of age, and applying standardized questionnaires via face-to-face interviews by trained field workers. The International Center for Equity in Health at the Federal University of Pelotas has been compiling all available surveys and reanalyzing selected indicators to improve comparability and enable inequality analyses. For the present study, we analyzed the most recent available survey available carried out since 2010.

Women aged 15–49 years are eligible to participate in these surveys. Our analysis was restricted to women that are married or in union because 18 of 91 surveys (including all surveys carried out in the Middle East and the North Africa regions) were limited to this group or to ever-married women. Our first indicator, demand for family planning satisfied with modern methods (DFPSm), was defined as the percentage of married women (or in union) in need of contraception who were using (or whose partner is using) any modern method of contraception. Women in need of contraception were those who are fecund and do not want to become pregnant within the next two years or who are unsure about whether or when they want to become pregnant [14, 15]. Pills, condoms (male and female), intrauterine devices (IUD), sterilization (male and female), injectables, implants, diaphragms, spermicidal agents (foam/jelly) and patches and emergency contraception were considered modern contraceptive methods. Emergency contraception account for a very small share of modern contraceptive use and was also included in our analyses considering that it is a product that interferes to prevent unintended pregnancies [18]. We included it in our analyses considering that it is a product that interferes with reproduction Our second indicator, institutional delivery, was defined as the percentage of the most recent live births in the two years preceding the survey that occurred in a health facility (in the private or public sector, regardless of the level of facility and health worker cadre who assisted with the birth). The analyses on DFPSm considered the woman’s age at the time of the survey, whereas the analyses on institutional delivery considered the woman’s age at the birth. Both age variables were categorized into seven 5 year-intervals (15–19; 20–24; 25–29; 30–34; 35–39; 40–44; 45–49 years). In DHS and MICS surveys, both woman’s current age and the date of birth of the last child, which is used to estimate the woman’s age at birth, are imputed for all women with completed interviews. Women with missing information on contraceptive use and place of most recent childbirth were considered as not having DFPSm and institutional delivery, respectively.

Descriptive analyses were initially designed to describe the coverage of the two indicators by women’s age group within each country with available data since 2010. Because it was not possible to describe in detail the individual patterns for each of the 91 countries, we attempted to develop a methodology for identifying age patterns using linear regression, testing for departure from linearity and applying fractional polynomials to the data. However, the large sample sizes in many surveys led to patterns that appeared on visual inspection to show smooth monotonic increases or declines with age whilst the statistical tests showed significant departures from linearity, or significant non-linear components in the fractional polynomial analyses (e.g. quadratic or cubic terms). For these reasons, we decided to identify the patterns based on visual inspection of the coverage levels by age group, rather than on the results of the regression analyses. Five distinct patterns were identified in the exploratory analyses; no other patterns were observed in the final analyses. The final visual inspection was performed by two authors independently (IS and FH). Of 89 age patterns available for each indicator, there was one country for which the two reviewers disagreed on the classification of DFPS, and five countries for institutional delivery. For these six cases (3.4% of all assessments), a third reviewer (FE) provided the final assessment. The patterns were (a) increasing coverage with woman’s age; (b) similar coverage in all age group; (c) U-shaped; (d) inverse U-shaped; and (e) declining coverage with woman’s age group. All country-specific graphs are included in the supplementary material for readers to refer to.

The frequency of the five patterns was examined according to UNICEF regions, World Bank income groups, and coverage at national level of the given indicator. UNICEF divides LMICs into seven regions: West & Central Africa, Eastern & Southern Africa, Middle East & North Africa, South Asia, East Asia & Pacific, Europe & Central Africa and Latin America & Caribbean. The World Bank income groups are based on the Gross National Income per capita, and countries are classified as low-income, lower-middle income and upper-middle income [19]. Countries were also categorized in terms of terciles of national coverage levels, calculated as described above.

## Results

We analyzed 91 surveys: 28 from low-income, 37 from lower-middle income and 26 from upper-middle income countries, which represent 82, 72 and 45% of all countries in each income group worldwide, respectively. Information needed to calculate DFPSm was not available in Jamaica (2011) and South Sudan (2010), whereas the institutional delivery indicator was not estimable for the surveys in Algeria 2012 (absence of woman’s age at birth) and St Lucia 2012 (due to small sample size, only 101 women with a birth in the last two years were included in the survey), totaling 89 countries analyzed for each. The description of all the countries, with the year of survey, coverage of DFPSm and institutional delivery at national level and the observed patterns of coverage by woman’s age are presented in Supplementary Table 1.

Figure 1 shows examples of each of the five patterns. The complete set of results is available in the Supplementary Figures 1–16. Age patterns are presented separately for each indicator and addressing the overall results followed by stratified analyses according to region, country income groupings and then by three levels of national coverage.

**Fig. 1:**
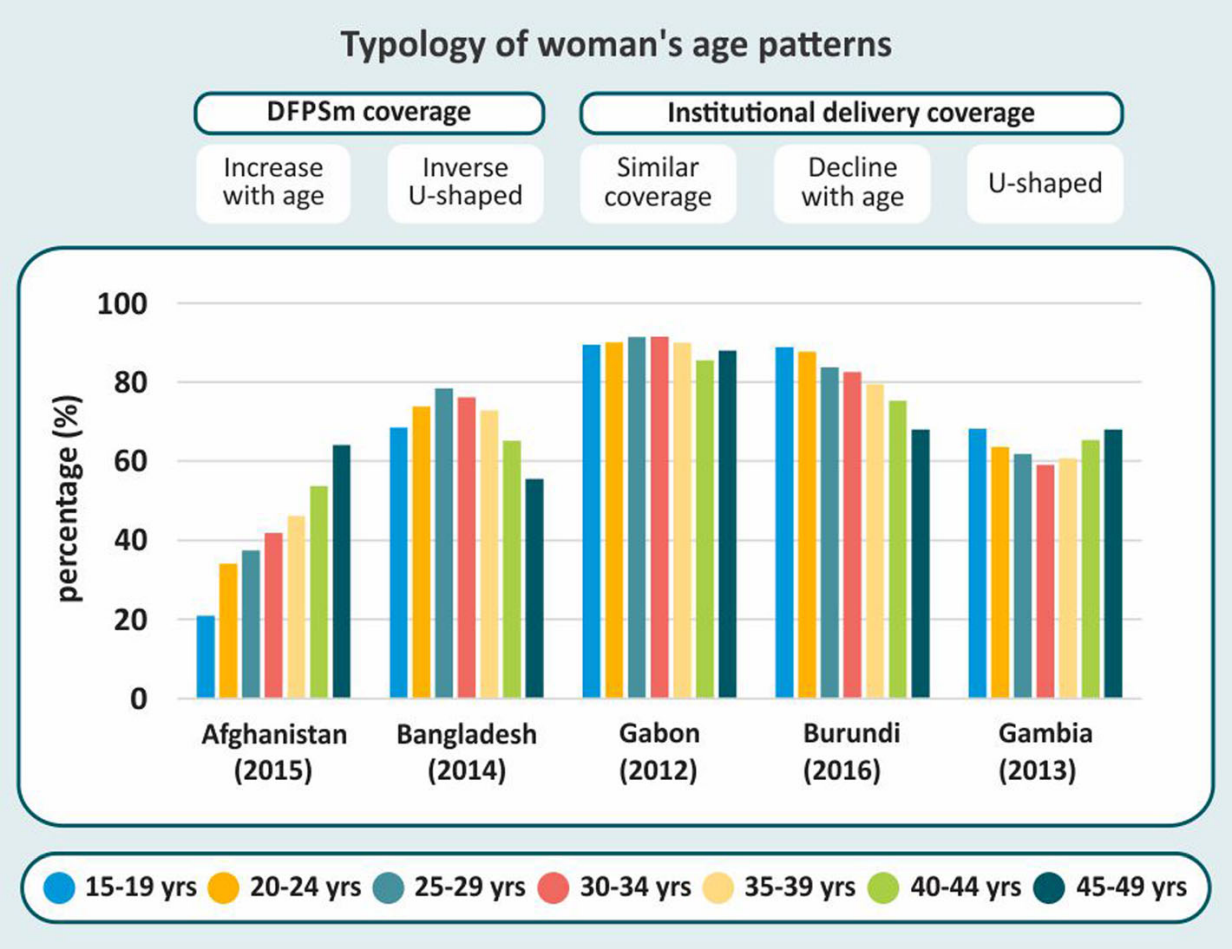
Typology of coverage by women’s age group (example countries). Note: No country showed U-shaped pattern for DFPSm and no country showed increasing coverage of institutional delivery with age

Table 1 shows that, for DFPSm coverage, the most frequent age patterns were inverse U-shaped (53%, 47 countries) and increasing coverage with age (41%, 36 countries). Only five countries (6%) presented a pattern of decline with age (Congo Brazzaville, Burundi, Ethiopia, Rwanda and Indonesia), only Peru showed similar coverage in all groups. None of the countries presented U-shaped patterns. Inverse U-shaped patterns were observed in seven of the eight (88%) countries in the Middle East & North Africa, in two thirds of the countries from the East Asia & Pacific, in seven countries in Europe & Central Asia (54%) and 13 in West & Central Africa (59%) regions. In contrast, most countries in South Asia (83%) and Latin America & Caribbean (63%) showed increasing coverage with age.

**Table 1:** Age patterns of DFPSm coverage according to UNICEF regions, World Bank country income group and tertiles of national coverage

	N	Increase with age N (%)	Inverse U-shaped N (%)	U-shaped N (%)	Decline with age N (%)	Similar coverage N (%)
**UNICEF region**						
West & Central Africa	22	8 (36.4)	13 (59.1)	0 (0.0)	1 (4.5)	0 (0.0)
Eastern & Southern Africa	15	4 (26.7)	8 (53.3)	0 (0.0)	3 (20.0)	0 (0.0)
Middle East & North Africa	8	1 (12.5)	7 (87.5)	0 (0.0)	0 (0.0)	0 (0.0)
South Asia	6	5 (83.3)	1 (16.7)	0 (0.0)	0 (0.0)	0 (0.0)
East Asia & Pacific	9	2 (22.2)	6 (66.7)	0 (0.0)	1 (11.1)	0 (0.0)
Europe & Central Asia	13	6 (46.2)	7 (53.8)	0 (0.0)	0 (0.0)	0 (0.0)
Latin America & Caribbean	16	10 (62.5)	5 (31.2)	0 (0.0)	0 (0.0)	1 (6.3)
**World Bank income group**						
Low income	27	11 (40.7)	13 (48.2)	0 (0.0)	3 (11.1)	0 (0.0)
Lower middle income	37	12 (32.4)	23 (62.2)	0 (0.0)	2 (11.2)	0 (0.0)
Upper middle income	25	13 (52.0)	11 (44.0)	0 (0.0)	0 (0.0)	1 (4.0)
**National coverage of DFPSm**						
First tertile (range: 11.3–38.9)	30	10 (33.3)	18 (60.0)	0 (0.0)	2 (6.7)	0 (0.0)
Second tertile (range: 39.4–72.5)	30	12 (40.0)	15 (50.0)	0 (0.0)	2 (6.7)	1 (3.3)
Third tertile (range: 72.6–92.9)	29	14 (48.3)	14 (48.3)	0 (0.0)	1 (3.4)	0 (0.0)
**Total**	**89**	**36 (40.5)**	**47 (52.8)**	**0 (0.0)**	**5 (5.6)**	**1 (1.1)**

Inverse U-shaped patterns for DFPSm was the most pronounced pattern among lower-middle income countries, where it was observed in two thirds of them (Table 1). Low- income and upper-middle income groups showed a more balanced distribution between increases with age and inverse U-shaped patterns. Finally, in the first and second tertiles of national coverage of DFPSm, inverse U-shaped was observed in more than half of countries, while increase with age and inverse U-shaped distributions were equally observed among countries in the third tertile (Table 1).

Table 2 shows the distribution of age patterns for institutional delivery. Declining coverage with age was the prevailing pattern (44%, 39 countries), followed by similar coverage across age groups (39%, 35 countries). Inverse U-shaped patterns were observed in 14 (16%) countries, and only the Gambia presented a U-shaped pattern. No country showed increasing coverage with age. In Europe & Central Asia and in the Middle East & North Africa, the most common pattern was no variation with age (85 and 86% of countries, respectively). In Eastern & Southern Africa and South Asia regions most countries showed declining coverage with age (80 and 67% respectively). The inverse U-shaped pattern, which was uncommon when analyzing all countries together, was present in 45% of countries in the East Asia & Pacific region.

**Table 2:** Age patterns of institutional delivery coverage according to UNICEF regions, World Bank country income group and tertiles of national coverage

	N	Increase with age N (%)	Inverse U-shaped N (%)	U-shaped N (%)	Decline with age N (%)	Similar coverage N (%)
**UNICEF region**						
West & Central Africa	23	0 (0.0)	4 (17.4)	1 (4.4)	11 (47.8)	7 (30.4)
Eastern & Southern Africa	15	0 (0.0)	1 (6.7)	0 (0.0)	13 (80.0)	2 (13.3)
Middle East & North Africa	7	0 (0.0)	0 (0.0)	0 (0.0)	1 (14.3)	6 (85.7)
South Asia	6	0 (0.0)	1 (16.7)	0 (0.0)	4 (66.6)	1 (16.7)
East Asia & Pacific	9	0 (0.0)	4 (44.5)	0 (0.0)	3 (33.3)	2 (22.2)
Europe & Central Asia	13	0 (0.0)	0 (0.0)	0 (0.0)	2 (15.4)	11 (84.6)
Latin America & Caribbean	16	0 (0.0)	3 (18.7)	0 (0.0)	7 (37.5)	7 (43.8)
**World Bank income group**						
Low income	28	0 (0.0)	3 (10.7)	1 (3.6)	20 (71.4)	4 (14.3)
Lower middle income	37	0 (0.0)	8 (21.6)	0 (0.0)	17 (46.0)	12 (32.4)
Upper middle income	24	0 (0.0)	2 (8.3)	0 (0.0)	3 (12.5)	19 (79.2)
**National coverage of Institutional delivery**						
First tertile (range: 11.7–63.6)	30	0 (0.0)	6 (20.0)	1 (3.3)	17 (56.7)	6 (20.0)
Second tertile (range: 64.5–91.5)	30	0 (0.0)	6 (20.0)	0 (0.0)	18 (60.0)	6 (20.0)
Third tertile (range: 92.3–99.9)	29	0 (0.0)	2 (6.9)	0 (0.0)	4 (13.8)	23 (79.3)
**Total**	**89**	**0 (0.0)**	**14 (15.7)**	**1 (1.1)**	**39 (43.8)**	**35 (39.3)**

There was marked variability in age patterns for institutional delivery by country income (Table 2). Most (79%) upper-middle income countries showed no variation by age group while most low-income countries showed declining coverage with age (71%). Similarly, most (79%) countries with high national coverage of institutional delivery showed no variation with age (Table 2) whilst 57% of those with the lowest national coverage showed a declining with age pattern.

We hypothesized that similar age pattens for both coverage indicators might be found within the same country. However, this was not the case, as only 9 out of 87 countries with both indicators had the same pattern for both (see Supplementary Table 2).

## Discussion

Our analysis of the coverage of DFPSm and institutional delivery across 91 LMICs revealed distinct age patterns for the two indicators. For family planning, the prevailing patterns were either an increase in coverage with older age or an inverse U-shaped pattern, suggesting that the need for modern contraceptives among currently married/cohabiting women is less satisfied among adolescents and also among older women. For institutional delivery, on the other hand, the prevailing patterns were either similar coverage across the age groups or a decrease in coverage with age. Hence, there is no indication that young women have lower coverage of institutional delivery. Even within the same country, the age patterns for DFPSm tended to be different from that observed for institutional delivery. Of the 87 countries with both indicators available, only 9 showed similar age pattern for both indicators.

In both prevailing patterns of DFPSm, adolescents had lower coverage than women in older age groups. Region of the world, country income and national DFPSm coverage were associated with age patterns. In countries with low or intermediate DFPSm coverage, inverse U-shaped patterns prevailed, whereas for high-coverage countries increases with age were as common as the inverse U-shaped pattern. Thus, it seems that as the national coverage increases, older women are reached and the inverse U-shaped pattern is gradually replaced by higher coverage among older women, and lower coverage among adolescents compared to the other age groups. It is interesting to note that – even at the 70% or more coverage levels found in the third tertile – similar coverage for all age groups is yet to be achieved in all the countries. South Asia and Latin America & Caribbean shown a markedly pattern of increase with age. In both regions, sterilization plays an important role in modern contraceptive use. It is observed a lower use of modern contraception where sterilization is not common, given the reduction of fertility and sexual activity.

Our findings for institutional delivery were markedly different to those observed for family planning. There were also marked differences by region and country income groups, that appeared to be mediated by national coverage levels: high-coverage countries tended to have similar coverage in all age groups, whereas most of the remaining countries tended to show declines with age. These findings suggest that increases in national coverage tend to be reflected in higher access to institutional delivery by older women, who would previously deliver at home. In fact, the top tertile of institutional delivery coverage is close to universal coverage, with more than 92% of the women being reached. These findings suggest that improvements in overall coverage of institutional delivery are less vulnerable to ongoing social inequities in health relative to that seen for national level improvements in family planning coverage. Most countries from East Asia & Pacific are in the first tertile of institutional delivery coverage and have lower levels of coverage among both older women and adolescents. In addition to the resistance of older women who had previously delivered at home, this pattern can be explained by the fact that most adolescent mothers in the region are from more vulnerable subgroups, such as those from lower socioeconomic levels and who live in rural areas.

In summary, both adolescent girls and – to a lesser extent – older women tended to show lower levels of demand for modern family planning satisfied in most countries, whereas for institutional delivery older women were more likely to be left behind. Pregnancy during adolescence has a major negative impact on women’s health and education [20], and children born to adolescent girls are at greater risk for neonatal mortality [21] so that family planning is particularly important in this age group. In many settings, child marriage is very common, and in this context, marriage often means motherhood as girls need to prove their fertility [22, 23]. We must address this and other social norms that may inhibit adolescents to reach health services in tandem with increasing access to sexual and reproductive health services and to contraception. In addition, women in late reproductive age, an often-forgotten population, are at increased risk for maternal mortality and those who become pregnant may be among the most vulnerable socially and biologically, so this is a concerning situation which must be tackled by public health initiatives [24, 25]. Strategies that have been shown to increase coverage include mobilization of political and community support, increased integration of services and, in some countries, public-private partnerships [26]. However, there is no place for one-size-fits-all approaches, as strategies for reaching universal coverage for these two interventions should be different, and, in addition, need to be tailored to specific country situations. Communication campaigns and other strategies to increase uptake across particular age groups may be needed for both interventions, but the content of the strategies will be very different for the two.

We did not have an a priori assumption that results would be similar for both indicators, for a number of reasons including the fact that national coverage levels tend to be higher for institutional delivery than for family planning, women’s desire for a given number of children, and that societal and religious norms seem more likely to affect contraception than delivery care. In addition, parity may be an important factor affecting coverage with both indicators, with younger women being less concerned than older women about unplanned pregnancies, and older women with previous healthy deliveries being less inclined to give birth in a hospital.

The present results contribute to the literature on intervention coverage by woman’s age, which has so far being mostly focused on differences among adolescents and older women, with the latter including ages 20–49 years [6–8]. In this paper, we used seven five-year age groups, which allowed us to find five different patterns of coverage by age. By doing so, we increased the granularity of age inequalities, and thus highlighted the vulnerability of older women (40 years or more), a group whose higher risk is not evident when the broad age group of 20–49 years is treated as a single category.

Evidence on the inverse association between age at first birth and institutional delivery had already documented for several low- and middle-income countries [27]. Globally, there is increasing emphasis on adolescents and even very young adolescents (10–14 years) in terms of comprehensive sexual education [28] and access to services, yet older women may have had little opportunity to receive this information when they were young. In perimenopause, there may be confusion regarding fertility, and this can compromise women’s health seeking for contraceptive use [24, 25, 29]. There may also be less investment in women in late reproductive age giving birth due to assumptions of they are already aware of the risks of late childbearing. Another possible explanation is that women in this age range are often multiparous, and may have had uneventful prior deliveries at home, and thus do not see the need for seeking care in an institution. In particular, recognition of increased risk for maternal mortality among older women giving birth, even when compared to adolescents, seems to be inadequately recognized. Poorest and highest fertility countries are most affected, but also poorest and rural women are likely the most affected within these countries [30].

Our analyses have limitations. Although data were analyzed for a large number of countries, these were not representative of all countries in the world: data were available for 82% of all low-income countries, 72% of lower-middle and 45% of upper-middle income countries. In addition, most surveys were carried out before 2015 in order to provide data for assessing success in terms of the Millennium Development Goals. Age heaping might be an issue, but we carried out sensitivity analyses with different age categories, which showed similar patterns in the extreme groups. It is also important to note that, according to the sampling methodology of the surveys, some groups of women were excluded. For institutional delivery analyses, all women with recent deliveries, regardless of marital status, except in a few countries (Afghanistan, Bangladesh, Egypt, Jordan and Pakistan) where the sample was restricted to married women. For family planning, we opted to include only women who were married or in union because 18 countries only had information for this group; for the 73 countries that also had information on unmarried women, only 11 showed a difference greater than 3% points between DFPSm coverage among women who were married or in union, and coverage among all sexually active women (results not shown). A final limitation is reliance on visual inspection of age patterns to derive a typology. This decision was taken after extensive attempts to use statistical approaches to identify such patterns; due to the large sample sizes in some surveys, statistical tests showed significant departures from linearity or from homogeneity, even when visual inspection showed monotonic increases or declines with age, or very similar coverage in all age groups. To address this limitation, we peer reviewed each pattern and the few inconsistencies in the typologies were reviewed by a third author. We also present in appendix (Supplementary Figures 1–16) the coverage by age in each country for the two coverage indicators, so that readers can assess the patterns. Future studies should attempt to deal with the above-mentioned limitations and seek for a better understand on locally-relevant mechanisms contributing to these age-related inequalities.

Parity was not taken into account in the present set of descriptive analyses, even though it likely affects coverage of DFPSm and institutional delivery [31, 32], and in addition is associated with socioeconomic position, education levels and other cultural characteristics. Further research is needed to elucidate the role of parity in the determinations of age patterns in coverage with RMNCH interventions.

Among the strengths of our analyses, these represent the most comprehensive overview so far on how coverage with two key interventions, both of which represent SDG goals or proxies for such goals, is affected by woman’s ages in a large number of LMICs. The standardized nature of the survey questionnaires, consistent indicator definitions and analytical methods also support the robustness of the present findings. Future studies should also investigate changes over time in age patterns, and expand the set of analyses to a broader range of reproductive and maternal health indicators. Characteristics of each health intervention and how they are delivered at population level may lead to different age patterns in coverage. Age-related inequalities should be routinely assessed, as is already the case for inequalities according to family wealth, education or sex.

## Conclusion

We identified five typologies of age-related patterns in coverage of two key reproductive and maternal health interventions. We found large inequalities in these indicators by age, which vary from country to country, and from one intervention to the other. Policy and programmatic approaches must be tailored to national patterns, and in most cases older women and adolescents will require special attention because they are at higher risk for maternal mortality and other poor obstetrical outcomes. Monitoring progress towards universal coverage needs to incorporate woman’s age as one of the key variables for stratification, in addition to more frequently studied stratifiers such as wealth or place of residence.

## Supplementary information

**Additional file 1: Table 1.** National coverage and Typology of woman’s age patterns of DFPSm and Institutional delivery. **Table 2.** Cross-tabulation of the 87 countries with information on both institutional delivery and DFPSm according to coverage patterns by woman’s age. Numbers in bold show similar age patterns for both coverage indicators within the same country. **Figures 1-8.** Demand for family planning satisfied with modern methods across woman’s age spectrum according to UNICEF regions. **Figures 9-16.** Institutional Delivery coverage across woman’s age spectrum according to UNICEF regions.

## Data Availability

All the datasets supporting the conclusions of this article are publicly available for download in the Demographic and Health Survey [https://dhsprogram.com/ data/available-datasets.cfm] and Multiple Indicator Cluster Survey [http://mics.unicef.org/surveys] websites.

## Abbreviations

DFPSm: Demand for family planning satisfied with modern methods
DHS: Demographic and Health Survey
MDG: Millennium Development Goal
MICS: Multiple Indicator Cluster Surveys
LMICs: Low- and middle-income countries
SDG: Sustainable Development Goals
