# Supplementary material for: Does women’s age matter in the SDGs era: coverage of demand for family planning satisfied with modern methods and institutional delivery in 91 low- and middle-income countries
Source: Reprod Health. 2020 Apr 19;17:55. doi: 10.1186/s12978-020-0903-6 (PMC7168879; doi:10.1186/s12978-020-0903-6)
Supplement: Supplementary file 1 — Additional file 1: Table 1. National coverage and Typology of woman’s age patterns of DFPSm and Institutional delivery. Table 2. Cross-tabulation of the 87 countries with information on both institutional delivery and DFPSm according to coverage patterns by woman’s age. Numbers in bold show similar age patterns for both coverage indicators within the same country. Figures 1-8. Demand for family planning satisfied with modern methods across woman’s age spectrum according to UNICEF regions. Figures 9-16. Institutional Delivery coverage across woman’s age spectrum according to UNICEF regions. [file 12978_2020_903_MOESM1_ESM.docx]

**
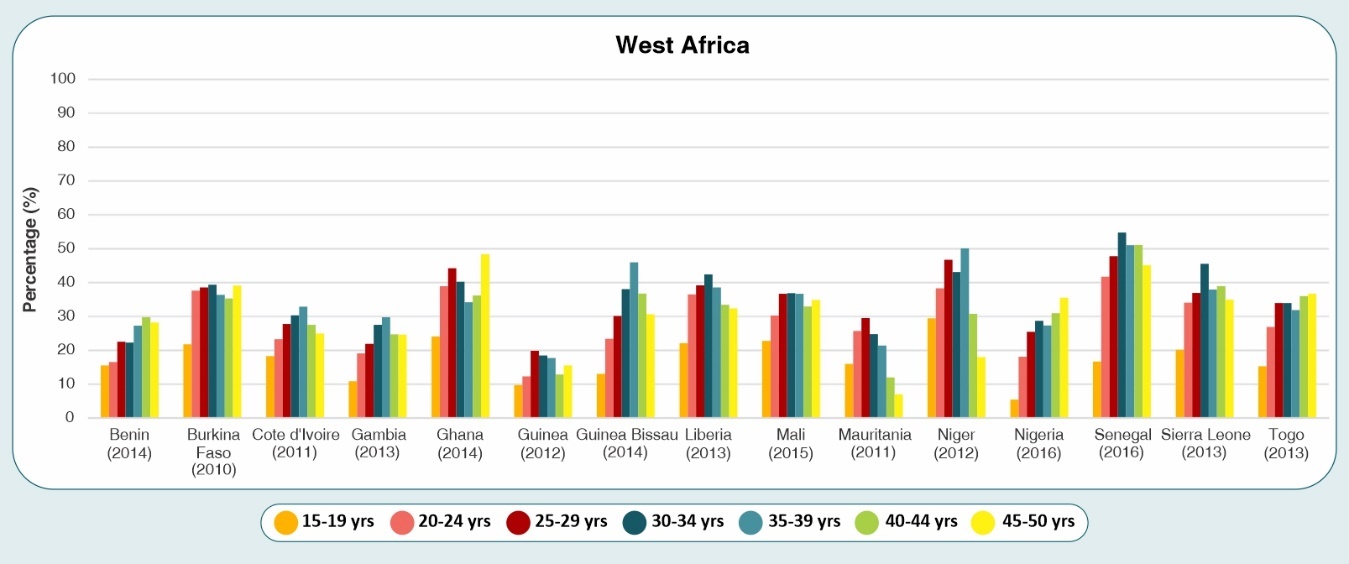
Does women’s age matter: coverage of demand for family planning satisfied with modern methods and institutional delivery in 91 low- and middle-income countries**

**Supplementary Table 1.** National coverage and Typology of woman’s age patterns of DFPSm and Institutional delivery.

| **Country (year)** | **Unicef regions** | **World Bank income group** | **DFPSm** | | **Institutional delivery** | |
| --- | --- | --- | --- | --- | --- | --- |
|  |  |  | **National coverage (%)** | **Typology of patterns** | **National coverage (%)** | **Typology of patterns** |
| Benin (2014) | West & Central Africa | Low | 23.3 | Increase with age | 87.0 | Similar coverage |
| Burkina Faso (2010) |  | Low | 36.6 | Increase with age | 66.3 | Decline with age |
| Cameroon (2014) |  | Lower-middle | 36.4 | Inverse U-shaped | 61.3 | Similar coverage |
| CAR (2010) |  | Low | 23.8 | Inverse U-shaped | 52.1 | Inverse U-shaped |
| Chad (2014) |  | Low | 14.0 | Increase with age | 21.9 | Similar coverage |
| Congo Brazzaville (2011) |  | Lower-middle | 29.1 | Decline with age | 91.5 | Similar coverage |
| Congo DR (2013) |  | Low | 14.8 | Increase with age | 79.9 | Decline with age |
| Cote dIvoire (2011) |  | Lower-middle | 25.6 | Inverse U-shaped | 57.4 | Similar coverage |
| Gabon (2012) |  | Upper-middle | 32.8 | Inverse U-shaped | 90.2 | Similar coverage |
| Gambia (2013) |  | Low | 23.8 | Inverse U-shaped | 62.6 | U-shaped |
| Ghana (2014) |  | Lower-middle | 38.4 | Increase with age | 73.1 | Inverse U-shaped |
| Guinea (2012) |  | Low | 11.3 | Inverse U-shaped | 40.6 | Decline with age |
| Guinea Bissau (2014) |  | Low | 34.4 | Inverse U-shaped | 44.0 | Decline with age |
| Liberia (2013) |  | Low | 37.0 | Inverse U-shaped | 55.8 | Decline with age |
| Mali (2015) |  | Low | 34.0 | Increase with age | 64.6 | Decline with age |
| Mauritania (2011) |  | Lower-middle | 21.7 | Inverse U-shaped | 64.5 | Inverse U-shaped |
| Niger (2012) |  | Low | 27.7 | Inverse U-shaped | 29.8 | Similar coverage |
| Nigeria (2016) |  | Lower-middle | 26.4 | Increase with age | 37.5 | Inverse U-shaped |
| Sao Tome and Principe (2014) |  | Lower-middle | 52.1 | Inverse U-shaped | 91.0 | Decline with age |
| Senegal (2016) |  | Low | 46.7 | Inverse U-shaped | 75.9 | Decline with age |
| Sierra Leone (2013) |  | Low | 35.3 | Inverse U-shaped | 54.4 | Decline with age |
| South Sudan (2010) |  | Low | NA | Not available | 11.7 | Decline with age |
| Togo (2013) |  | Low | 32.2 | Increase with age | 72.8 | Decline with age |
| Angola (2015) | Eastern & Southern Africa | Lower-middle | 23.8 | Inverse U-shaped | 45.6 | Decline with age |
| Burundi (2016) |  | Low | 38.3 | Decline with age | 83.9 | Decline with age |
| Comoros (2012) |  | Low | 25.8 | Increase with age | 76.1 | Inverse U-shaped |
| Eswatini (2014) |  | Lower-middle | 85.3 | Inverse U-shaped | 87.7 | Similar coverage |
| Ethiopia (2016) |  | Low | 60.2 | Decline with age | 26.2 | Decline with age |
| Kenya (2014) |  | Lower-middle | 70.5 | Inverse U-shaped | 61.5 | Decline with age |
| Lesotho (2014) |  | Lower-middle | 76.1 | Inverse U-shaped | 76.5 | Decline with age |
| Malawi (2015) |  | Low | 74.4 | Increase with age | 91.4 | Decline with age |
| Mozambique (2011) |  | Low | 31.4 | Inverse U-shaped | 54.8 | Decline with age |
| Namibia (2013) |  | Upper-middle | 74.7 | Increase with age | 87.7 | Decline with age |
| Rwanda (2014) |  | Low | 64.3 | Decline with age | 90.7 | Decline with age |
| Tanzania (2015) |  | Low | 52.1 | Increase with age | 62.6 | Decline with age |
| Uganda (2016) |  | Low | 49.7 | Inverse U-shaped | 73.4 | Decline with age |
| Zambia (2013) |  | Lower-middle | 62.4 | Inverse U-shaped | 67.6 | Decline with age |
| Zimbabwe (2015) |  | Low | 84.9 | Inverse U-shaped | 77.0 | Inverse U-shaped |
| Algeria (2012) | Middle East & North Africa | Upper-middle | 80.7 | Inverse U-shaped | - | Not available |
| Egypt (2014) |  | Lower-middle | 80.0 | Inverse U-shaped | 86.7 | Similar coverage |
| Iraq (2011) |  | Upper-middle | 59.3 | Increase with age | 76.6 | Similar coverage |
| Jordan (2012) |  | Upper-middle | 55.9 | Inverse U-shaped | 98.8 | Similar coverage |
| State of Palestine (2014) |  | Lower-middle | 69.1 | Inverse U-shaped | 99.3 | Similar coverage |
| Sudan (2014) |  | Lower-middle | 31.6 | Inverse U-shaped | 27.7 | Similar coverage |
| Tunisia (2011) |  | Upper-middle | 74.3 | Inverse U-shaped | 98.5 | Similar coverage |
| Yemen (2013) |  | Lower-middle | 40.5 | Inverse U-shaped | 30.7 | Decline with age |
| Afghanistan (2015) | South Asia | Low | 39.4 | Increase with age | 48.3 | Similar coverage |
| Bangladesh (2014) |  | Lower-middle | 72.6 | Inverse U-shaped | 37.6 | Decline with age |
| Bhutan (2010) |  | Lower-middle | 85.8 | Increase with age | 63.1 | Inverse U-shaped |
| India (2015) |  | Lower-middle | 71.8 | Increase with age | 78.9 | Decline with age |
| Nepal (2016) |  | Low | 56.0 | Increase with age | 57.4 | Decline with age |
| Pakistan (2012) |  | Lower-middle | 44.0 | Increase with age | 48.2 | Decline with age |
| Cambodia (2014) | East Asia & Pacific | Lower-middle | 56.1 | Inverse U-shaped | 83.2 | Decline with age |
| Indonesia (2012) |  | Lower-middle | 78.9 | Decline with age | 63.6 | Inverse U-shaped |
| Lao (2011) |  | Lower-middle | 68.6 | Inverse U-shaped | 37.5 | Decline with age |
| Mongolia (2013) |  | Lower-middle | 74.9 | Inverse U-shaped | 97.1 | Similar coverage |
| Myanmar (2015) |  | Lower-middle | 74.8 | Inverse U-shaped | 37.1 | Inverse U-shaped |
| Philippines (2013) |  | Lower-middle | 50.8 | Inverse U-shaped | 61.1 | Decline with age |
| Thailand (2012) |  | Upper-middle | 92.9 | Increase with age | 99.6 | Similar coverage |
| Timor Leste (2016) |  | Lower-middle | 45.8 | Increase with age | 48.5 | Inverse U-shaped |
| Vietnam (2013) |  | Lower-middle | 73.2 | Inverse U-shaped | 93.6 | Inverse U-shaped |
| Armenia (2015) | Europe & Central Asia | Lower-middle | 38.9 | Inverse U-shaped | 99.8 | Similar coverage |
| Belarus (2012) |  | Upper-middle | 76.0 | Inverse U-shaped | 99.9 | Similar coverage |
| Bosnia and Herzegovina (2011) |  | Upper-middle | 23.0 | Inverse U-shaped | 99.7 | Similar coverage |
| Kazakhstan (2015) |  | Upper-middle | 85.2 | Increase with age | 99.3 | Similar coverage |
| Kosovo (2013) |  | Lower-middle | 21.0 | Increase with age | 99.0 | Similar coverage |
| Kyrgyzstan (2014) |  | Lower-middle | 68.7 | Increase with age | 98.3 | Similar coverage |
| Macedonia (2011) |  | Upper-middle | 26.7 | Inverse U-shaped | 98.4 | Similar coverage |
| Moldova (2012) |  | Lower-middle | 66.5 | Inverse U-shaped | 98.9 | Decline with age |
| Montenegro (2013) |  | Upper-middle | 40.5 | Increase with age | 99.0 | Similar coverage |
| Serbia (2014) |  | Upper-middle | 31.5 | Inverse U-shaped | 98.3 | Similar coverage |
| Tajikistan (2012) |  | Low | 50.6 | Inverse U-shaped | 76.5 | Decline with age |
| Turkmenistan (2015) |  | Upper-middle | 78.5 | Increase with age | 99.5 | Similar coverage |
| Ukraine (2012) |  | Lower-middle | 69.4 | Increase with age | 98.9 | Similar coverage |
| Belize (2011) | Latin America & Caribbean | Lower-middle | 73.1 | Increase with age | 93.8 | Decline with age |
| Colombia (2015) |  | Upper-middle | 86.5 | Inverse U-shaped | 96.9 | Inverse U-shaped |
| Costa Rica (2011) |  | Upper-middle | 89.3 | Increase with age | 98.1 | Similar coverage |
| Cuba (2014) |  | Upper-middle | 89.7 | Increase with age | 99.0 | Similar coverage |
| Dominican Republic (2014) |  | Upper-middle | 85.2 | Increase with age | 97.9 | Similar coverage |
| El Salvador (2014) |  | Lower-middle | 84.8 | Increase with age | 97.5 | Decline with age |
| Guatemala (2014) |  | Lower-middle | 65.3 | Increase with age | 65.0 | Decline with age |
| Guyana (2014) |  | Upper-middle | 52.4 | Increase with age | 92.7 | Decline with age |
| Haiti (2012) |  | Low | 44.2 | Inverse U-shaped | 35.9 | Decline with age |
| Honduras (2011) |  | Lower-middle | 76.0 | Inverse U-shaped | 82.7 | Decline with age |
| Jamaica (2011) |  | Upper-middle | NA | Not available | 98.6 | Similar coverage |
| Mexico (2015) |  | Upper-middle | 86.1 | Increase with age | 96.9 | Similar coverage |
| Panama (2013) |  | Upper-middle | 76.4 | Increase with age | 91.2 | Inverse U-shaped |
| Paraguay (2016) |  | Upper-middle | 86.4 | Inverse U-shaped | 93.2 | Similar coverage |
| Peru (2012) |  | Upper-middle | 61.0 | Similar coverage | 84.7 | Inverse U-shaped |
| St Lucia (2012) |  | Upper-middle | 72.5 | Inverse U-shaped | 100.0 | Not available |
| Suriname (2010) |  | Upper-middle | 73.2 | Increase with age | 92.3 | Similar coverage |

**Supplementary Table 2.** Cross-tabulation of the 87 countries with information on both institutional delivery and DFPSm according to coverage patterns by woman’s age. Numbers in bold show similar age patterns for both coverage indicators within the same country.

|  | | **Institutional delivery pattern** | | | | |
| --- | --- | --- | --- | --- | --- | --- |
|  |  | Increase with age | Inverse U shaped | U-shaped | Decline with age | Similar coverage |
| **DFPSm pattern** | Increase with age | **0** | 6 | 0 | 14 | 16 |
|  | Inverse U-shaped | 0 | **6** | 1 | 21 | 17 |
|  | U-shaped | 0 | 0 | **0** | 0 | 0 |
|  | Decline with age | 0 | 1 | 0 | **3** | 1 |
|  | Similar coverage | 0 | 1 | 0 | 0 | **0** |

**Supplementary Figures**

**Figures 1 – 8.** Demand for family planning satisfied with modern methods across woman’s age spectrum according to UNICEF regions.

**Supplementary Figure 1.**

**
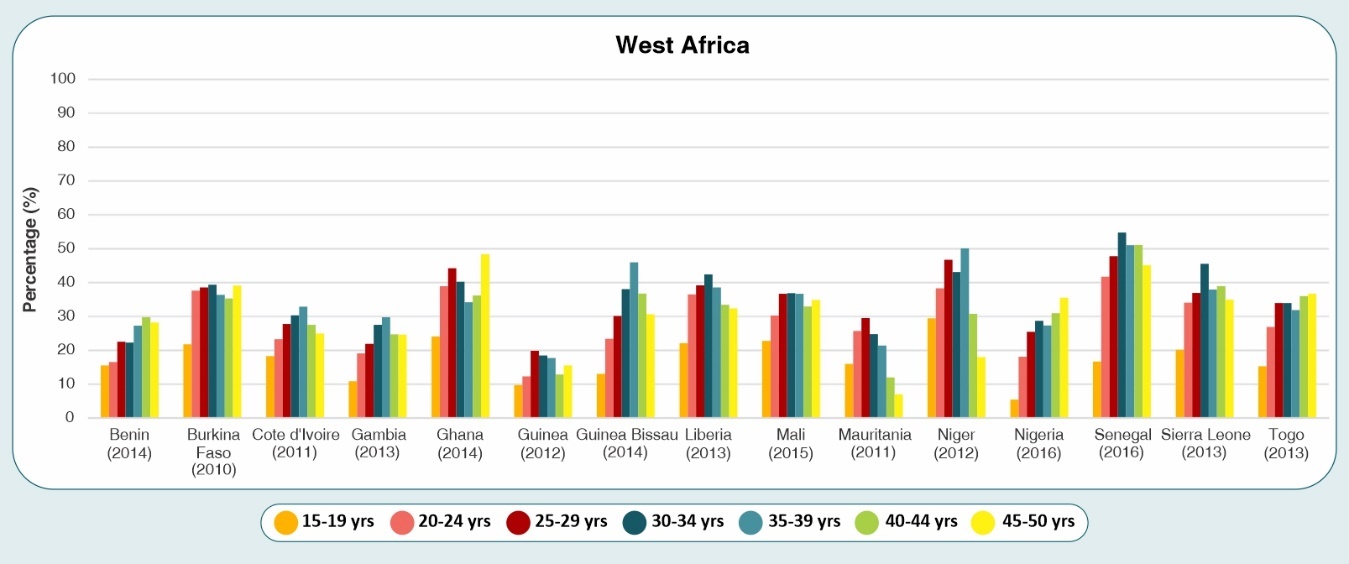
**

**Supplementary Figure 2.**

**
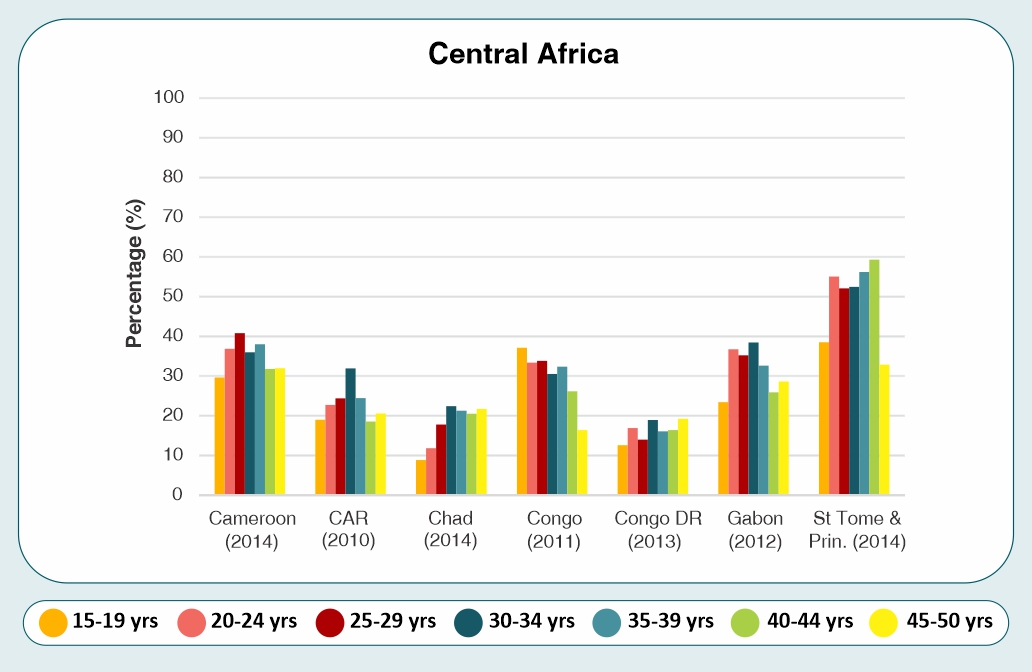
**

**Supplementary Figure 3.**

**
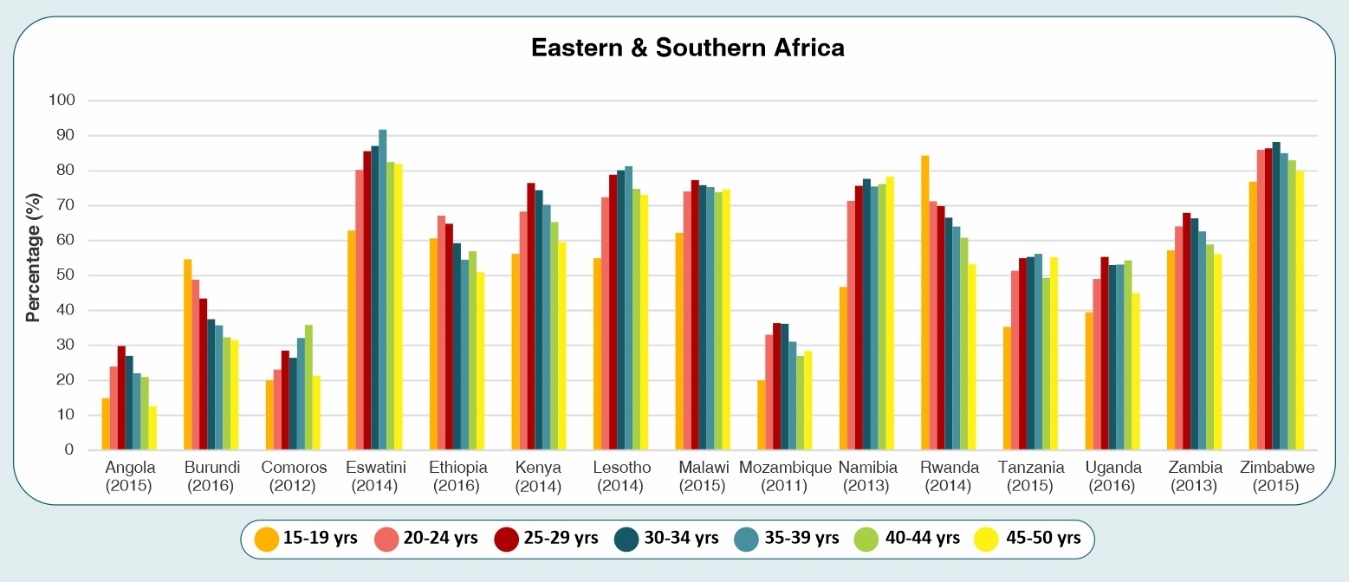
**

**Supplementary Figure 4.**

**
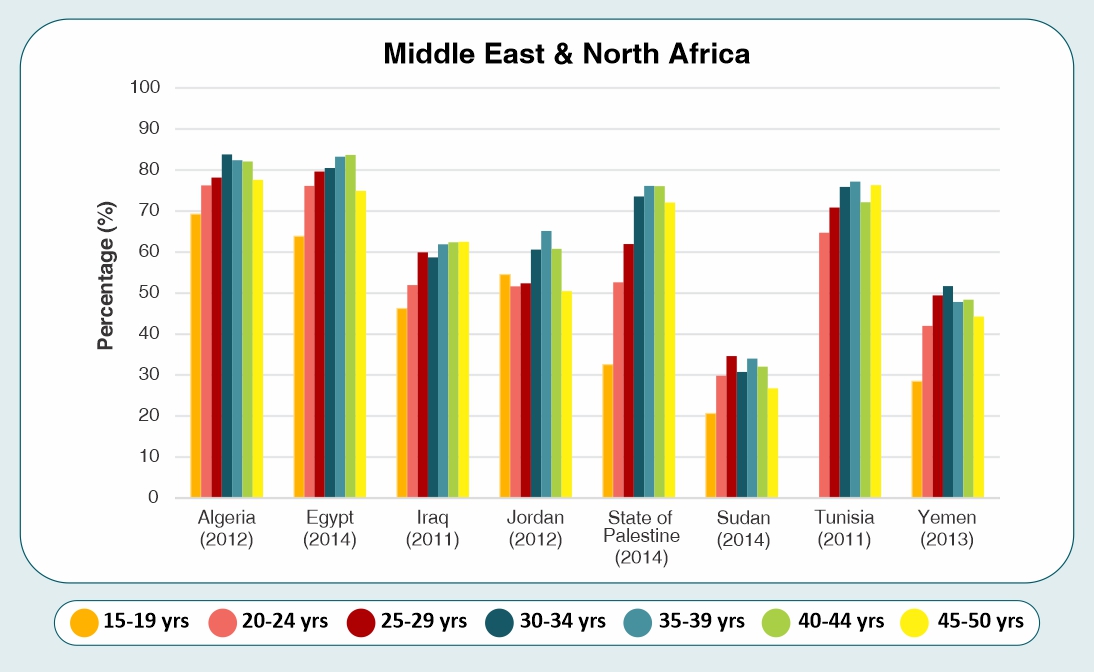
**

**Supplementary Figure 5.
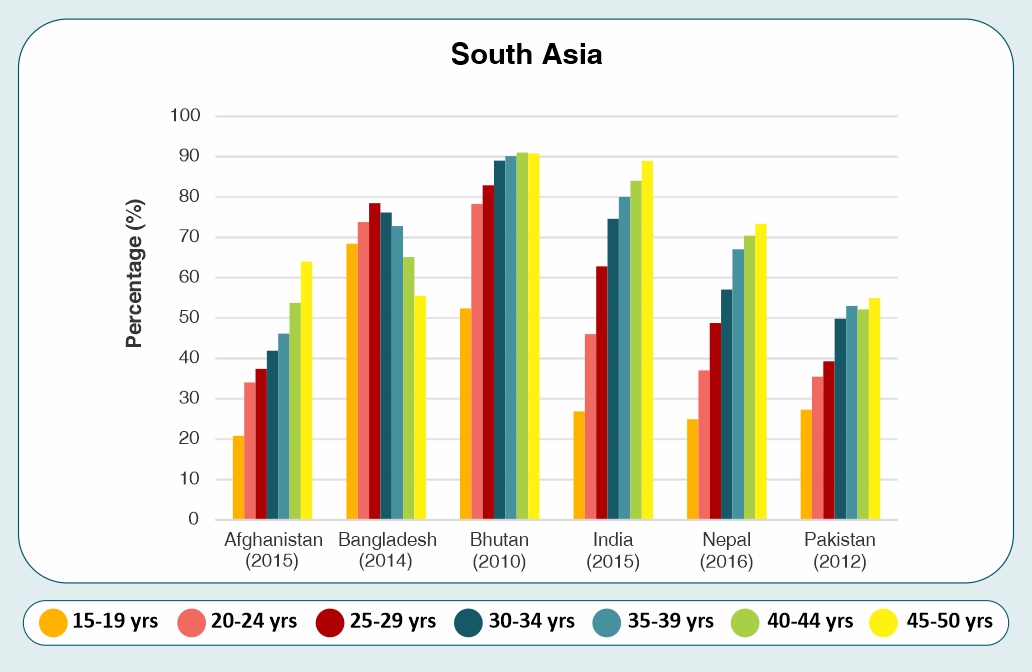
**

**Supplementary Figure 6.**

**
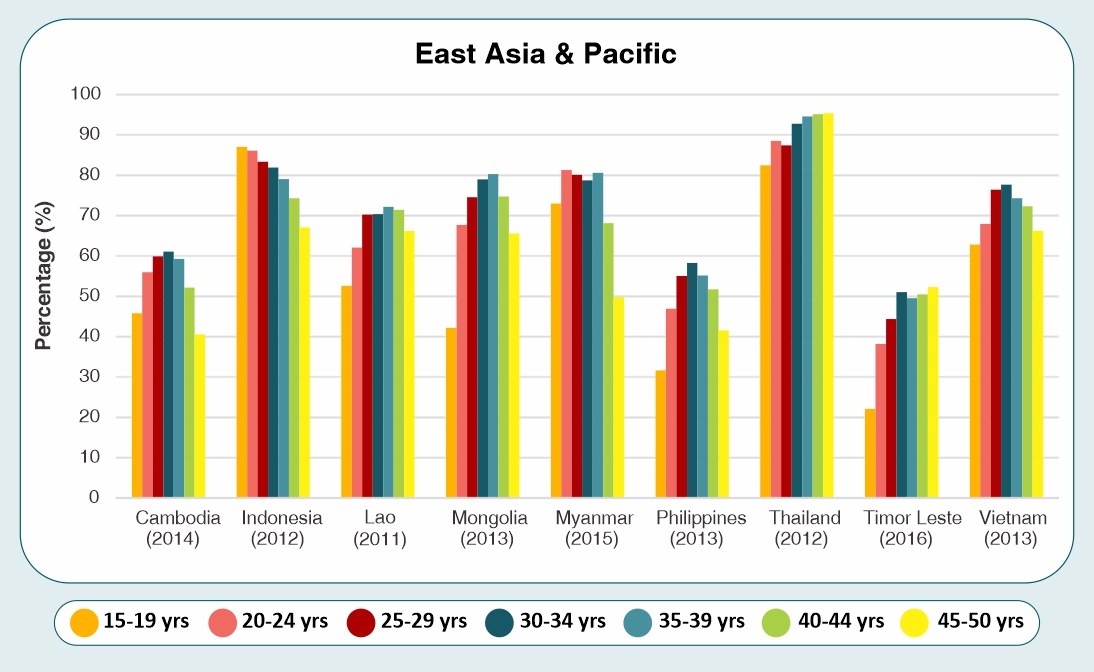
**

**Supplementary Figure 7.**

**
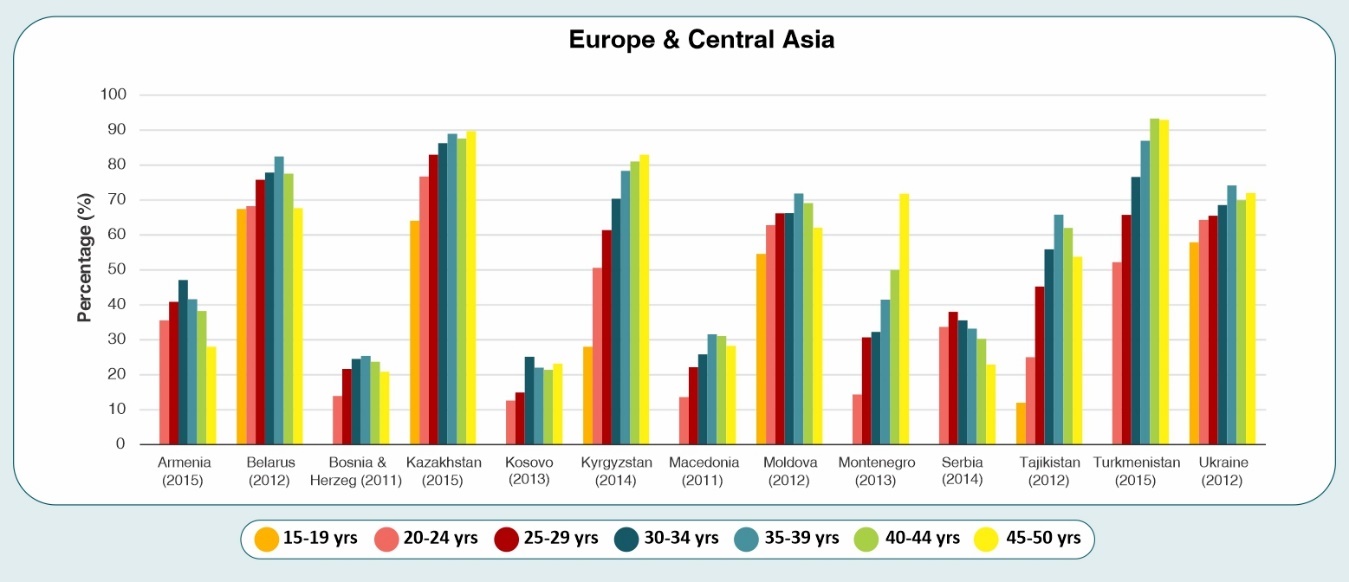
**

**Supplementary Figure 8.**

**
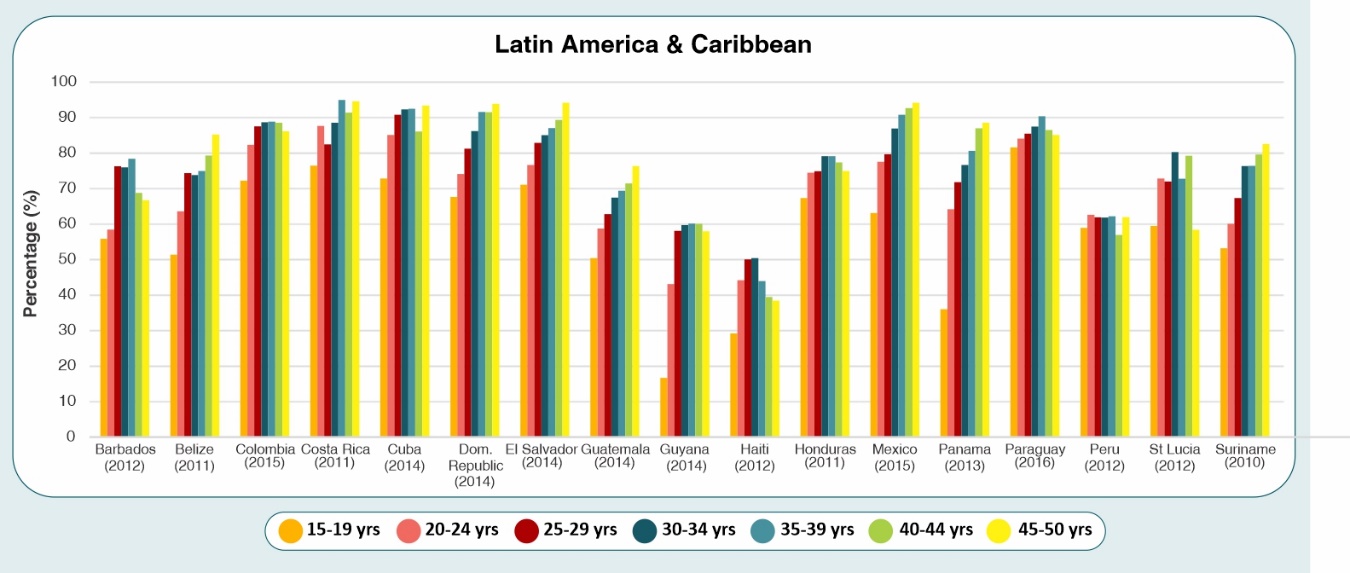
**

**Figures 9 – 16.** Institutional Delivery coverage across woman’s age spectrum according to UNICEF regions.

**Supplementary Figure 9.**

**
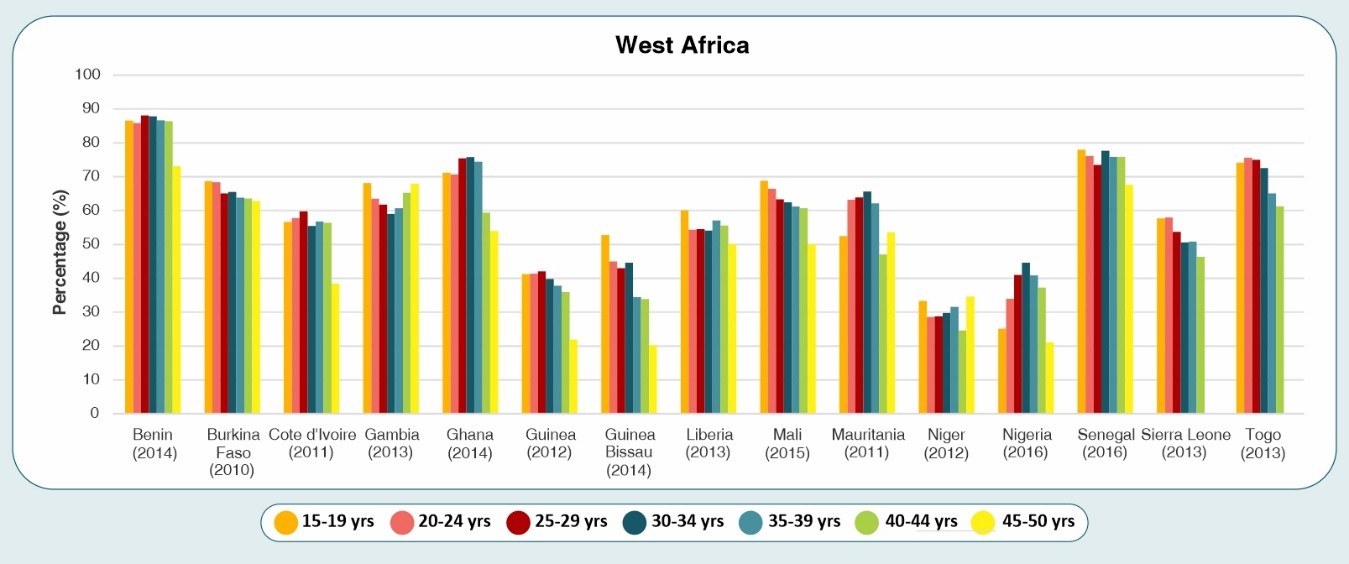
**

**Supplementary Figure 10.**

**
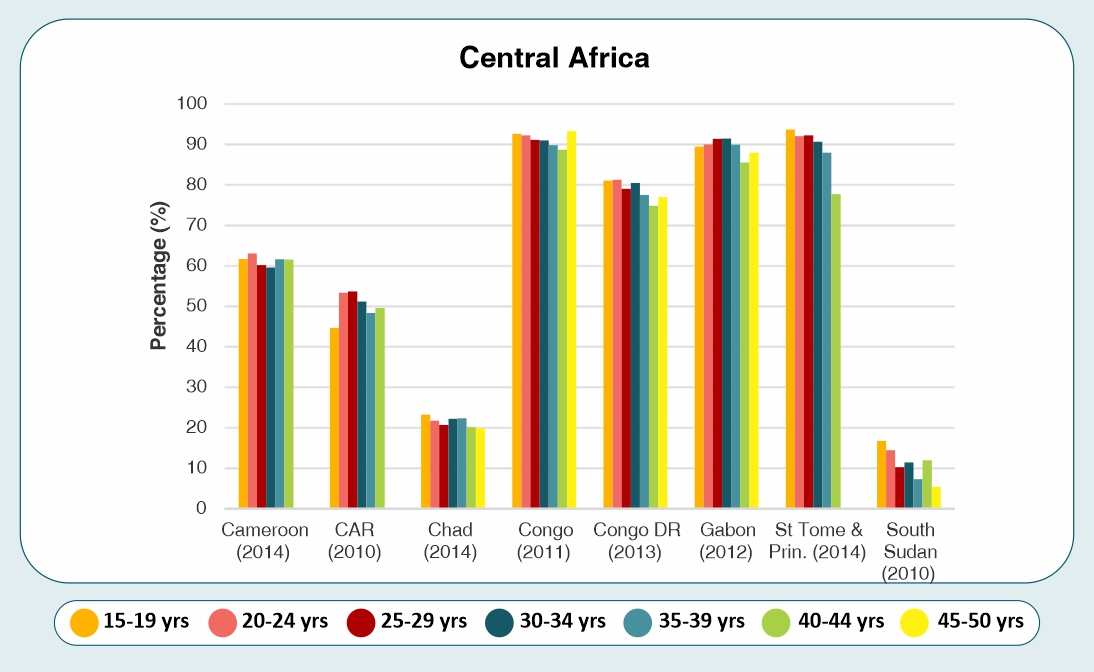
**

**Supplementary Figure 11.**

**
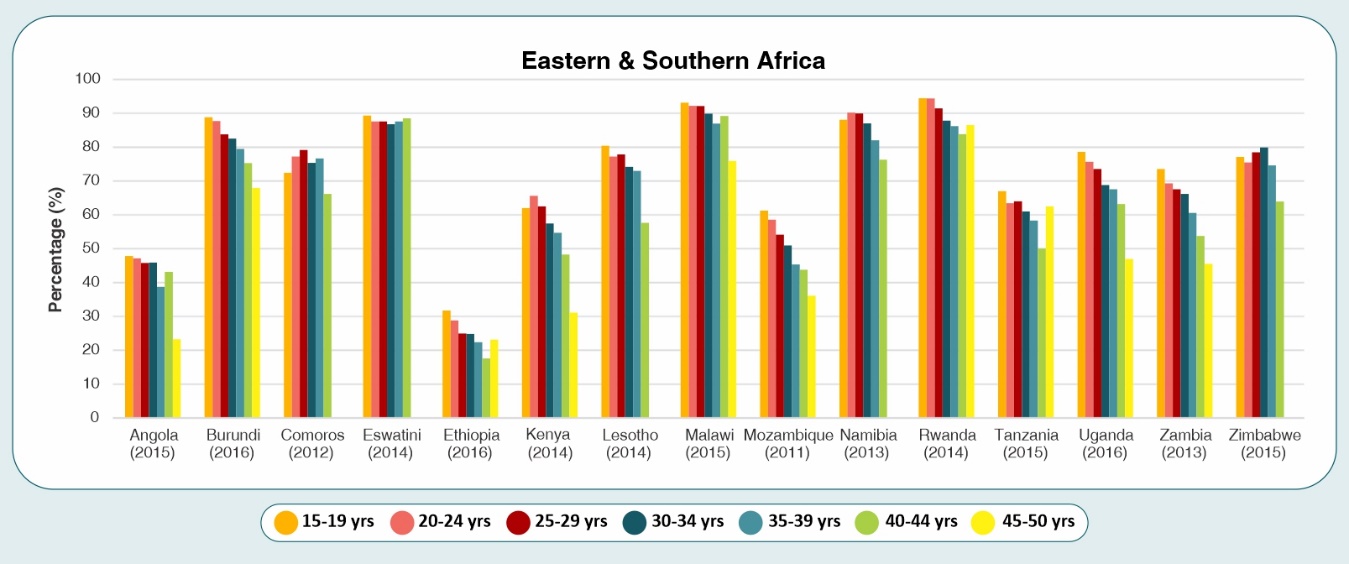
**

**Supplementary Figure 12.**

**
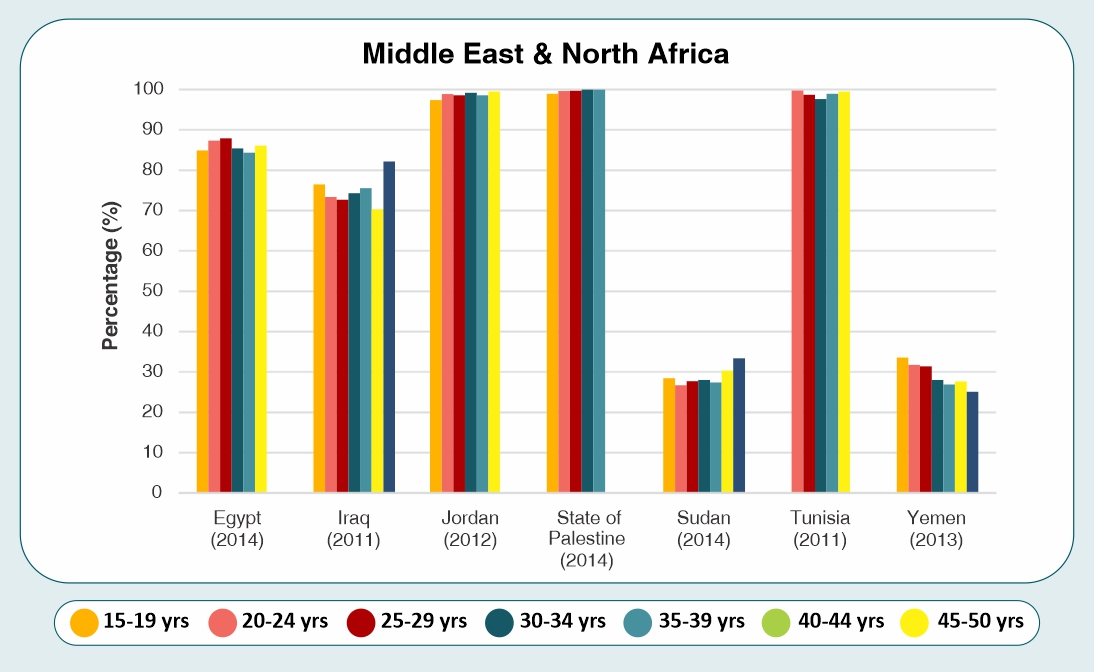
**

**Supplementary Figure 13.**

**
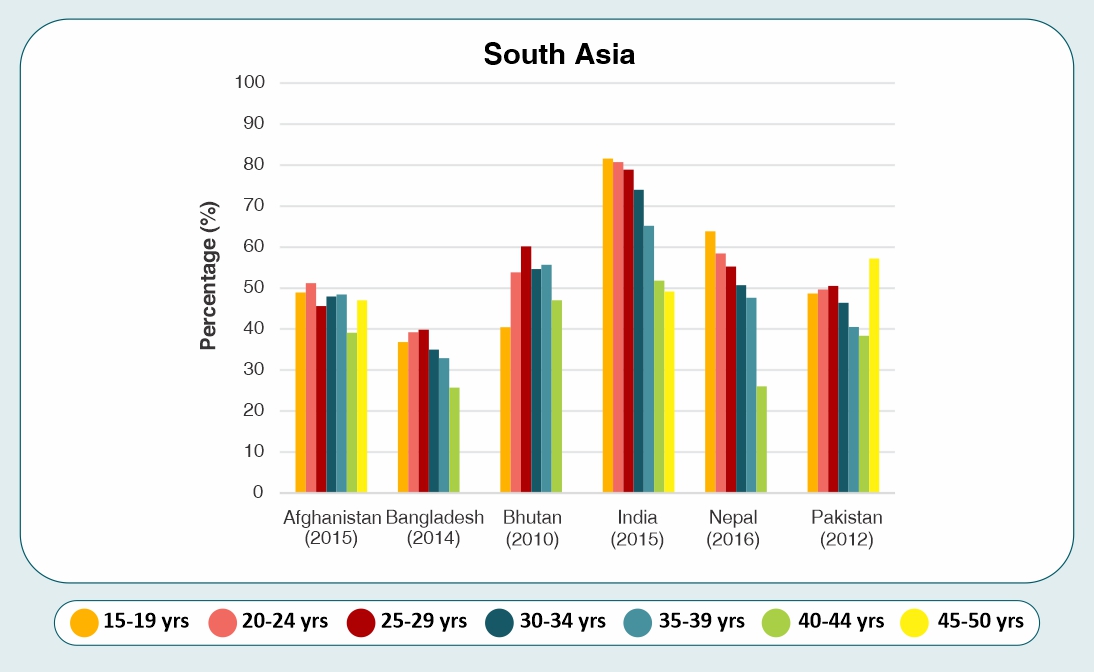
**

**Supplementary Figure 14.**

**
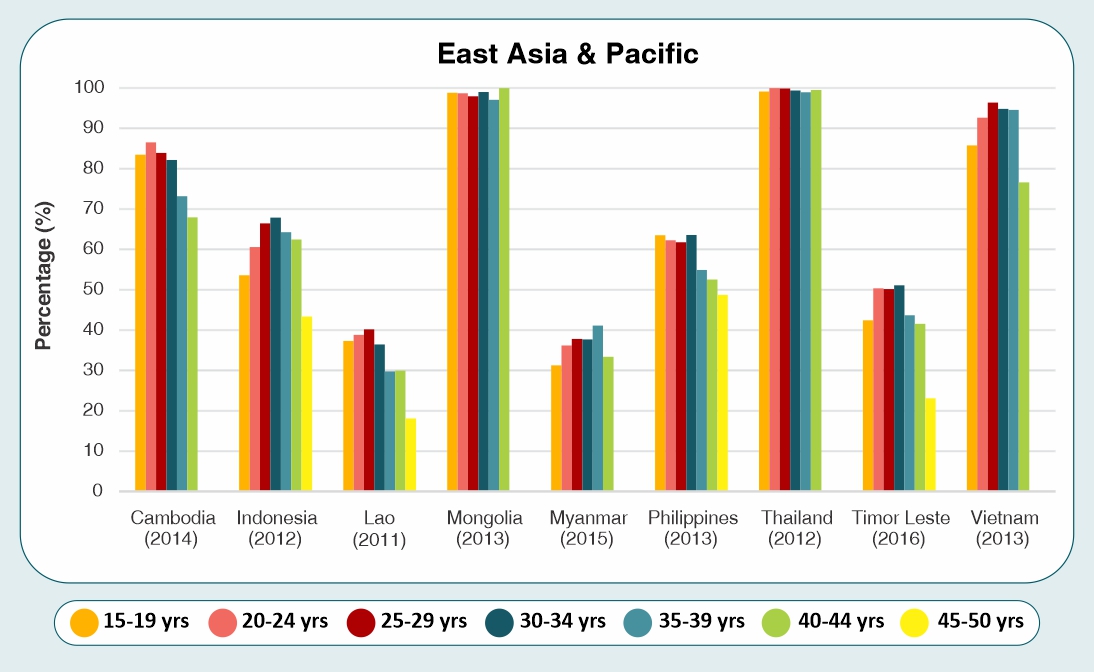
**

**Supplementary Figure 15.**

**
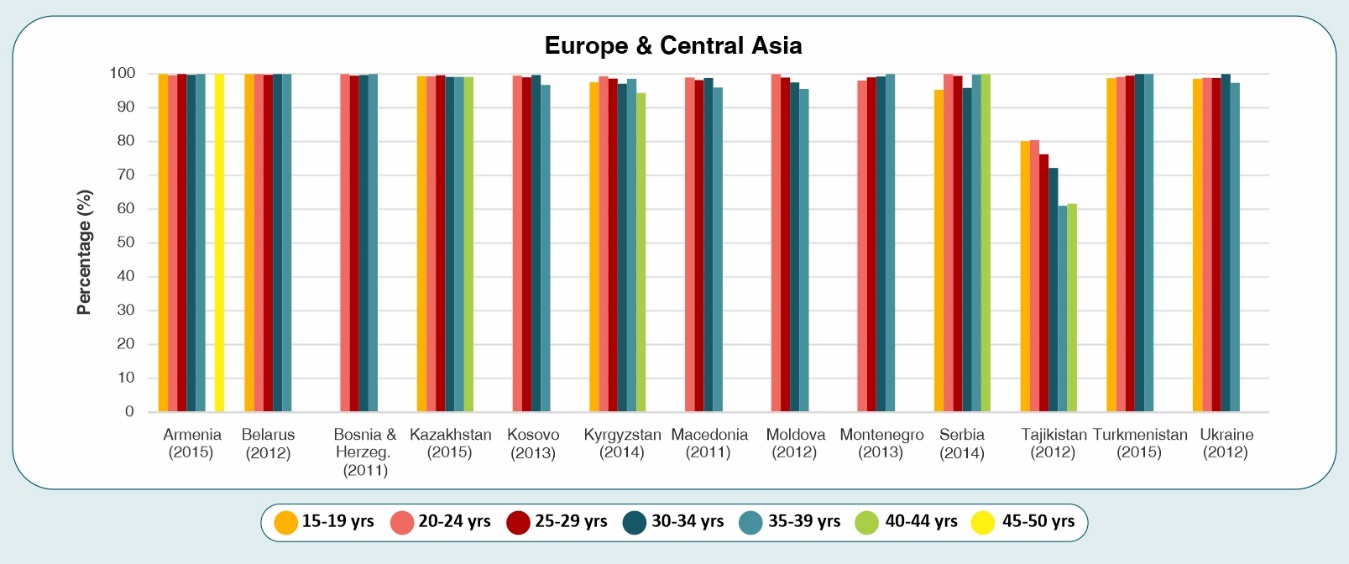
**

**Supplementary Figure 16.**

**
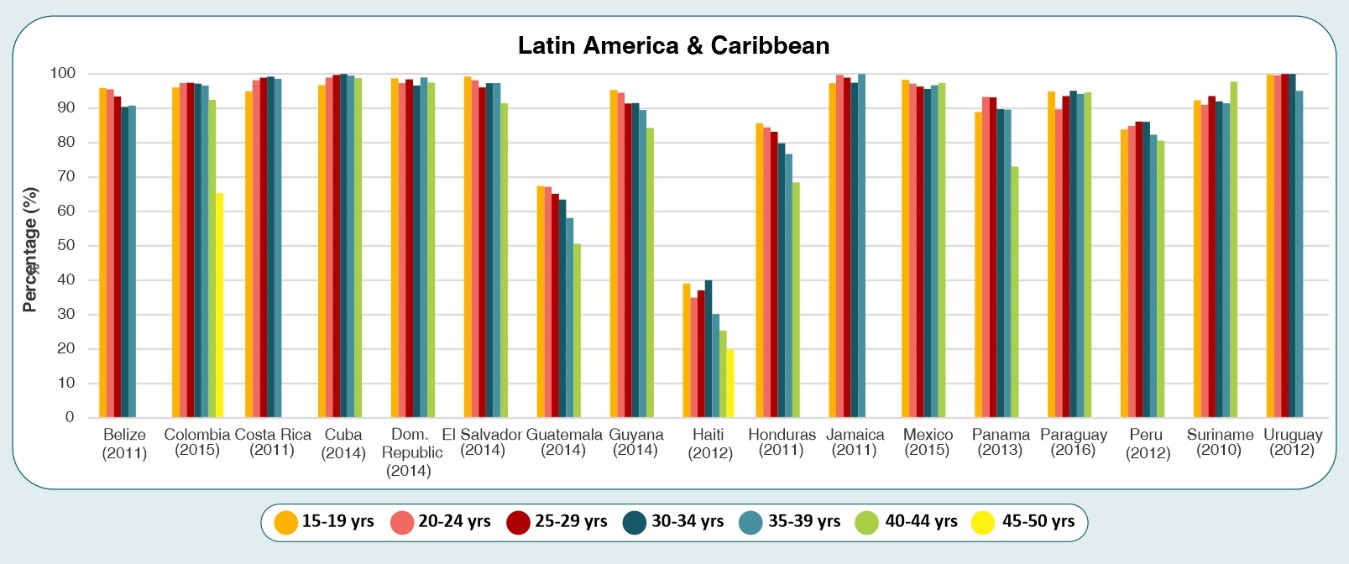
**
